# High Transferability of Neutralizing Antibodies against SARS-CoV-2 to Umbilical Cord Blood in Pregnant Women Vaccinated with BNT162b2 XBB.1.5: A Retrospective Cohort Study

**DOI:** 10.3390/idr16030036

**Published:** 2024-05-27

**Authors:** Takuma Hayashi, Kenji Sano, Ikuo Konishi

**Affiliations:** 1 Cancer Medicine, National Hospital Organization Kyoto Medical Center, 1-1, Fukakusa Mukaihatake, Fushimi-ku, Kyoto-City 612-8555, Kyoto, Japan; 2 Medical R&D Promotion Project, The Japan Agency for Medical Research and Development (AMED), Chuo-ku, Tokyo 103-0022, Japan; 3 School of Medicine Hospital, Shinshu University, Matumoto-City 390-0877, Nagano, Japan; 4 School of Medicine, Kyoto University, Kyoto-City 606-8507, Kyoto, Japan

**Keywords:** SARS-CoV-2, Spike glycoprotein, BNT162b2 XBB.1.5 vaccine, IgG, IgA, COVID-19

## Abstract

Background: Coronavirus disease 2019 (COVID-19) can lead to severe respiratory illness, rapid disease progression, and higher rates of intensive care unit admission in pregnant women. Infection during pregnancy is associated with an increased risk of preterm delivery, cesarean section, fetal dysfunction, preeclampsia, and perinatal death. Vertical transmission of severe acute respiratory syndrome coronavirus 2 (SARS-CoV-2) from pregnant women to their fetuses has also been observed. Although severe infections in neonates and infants are rare, newborns can experience serious consequences from COVID-19 due to their suboptimal humoral immune system protection. The amino acids in the structural proteins of SARS-CoV-2 are constantly mutating. Since around January 2023, COVID-19, caused by omicron-type SARS-CoV-2 variants, has been prevalent globally. These variants can evade the immune response triggered by traditional mRNA-based COVID-19 vaccines, such as BNT162b2. Therefore, vaccination with BNT162b2 XBB.1.5, which provides protection against omicron-type SARS-CoV-2 variants, is recommended. Methods: This retrospective cohort study included 148 pregnant women who received the BNT162b2 XBB.1.5 vaccine at 30 partner medical institutions from September 2023 to January 2024. We examined the titers of anti-spike glycoprotein SARS-CoV-2 immunoglobin G (IgG) and IgA in the blood and umbilical cord blood obtained from the participants using ELISA. Findings: Anti-spike glycoprotein SARS-CoV-2 IgG and IgA titers were highest in the blood and cord blood at late gestational age (28–34 weeks). No serious side effects or adverse events were observed in either the pregnant women or their newborns. Interpretation: Pregnant women who received the BNT162b2 XBB.1.5 vaccine during gestational weeks 28 to 34 had the highest titers of anti-omicron SARS-CoV-2 variant antibodies in their blood. Moreover, these antibodies were transferred to their umbilical cord blood. To validate our findings, large cohort clinical studies involving numerous pregnant women are warranted. Funding: This study was funded by Grants-in-Aid for Scientific Research from the Japan Society for the Promotion of Science (JSPS) and Grants-in-Aid for Medical Research from the Japan Agency for Medical Research and Development (AMED).

## 1. Introduction

Severe acute respiratory syndrome coronavirus 2 (SARS-CoV-2) undergoes frequent mutations, leading to the emergence of new variants that can spread rapidly, and there have been many variants throughout the duration of the COVID-19 pandemic. In 2023, the omicron-type SARS-CoV-2 XBB.1.5 subvariant, distinguished by the Ser486Pro mutation within the omicron-type SARS-CoV-2 XBB.1 lineage, began spreading mainly in North America [1]. In the United States, the omicron-type SARS-CoV-2 BA.5 subvariant was the predominant strain of coronavirus infections since the onset of the coronavirus disease 2019 (COVID-19) pandemic in 2019 until around November 2022. However, from January 2023 onward, the BQ.1 and BQ.1.1 subvariants became prevalent. Subsequently, the infection of the omicron-type SARS-CoV-2 XBB.1.5 subvariant spread rapidly [2].

Vaccination is the most effective prevention against many viral infections. However, with each new virus variant, the preventive effects and antiviral antibodies generated by vaccination tend to diminish [3]. An alarming issue of SARS-CoV-2 infection is the aggravation in elderly individuals and newborns, who often show diminished type 1 immune responses. Particularly, the transfer of vaccine-induced anti-SARS-CoV-2 antibodies from pregnant women through the umbilical cord blood and/or breast milk to the fetus may be effective against neonatal COVID-19 [4,5].

COVID-19 vaccination (Pfizer/BioNTech; BNT162b2) during pregnancy stimulates the production of functional anti-SARS-CoV-2 spike glycoprotein immunoglobulin G (IgG) and IgA in the circulation of pregnant women. These antibodies are also detectable in the umbilical cord blood at the time of birth. The anti-SARS-CoV-2 spike glycoprotein IgG and IgA generated in the mother’s body through BNT162b2 vaccination during pregnancy may confer protection to newborns and infants against SARS-CoV-2 infection [6,7,8,9]. The titer of anti-SARS-CoV-2 spike glycoprotein IgG and IgA in cord blood correlates with the levels in maternal serum, with the highest concentrations observed after vaccination in the late second and early third trimesters [7,8,9]. XBB.1.5, a variant of the Omicron strain of SARS-CoV-2, shows high resistance to the immunity induced by BNT162b2 vaccination or SARS-CoV-2 infection. Consequently, Pfizer/BioNTech has obtained approval in many countries to produce and distribute a vaccine (BNT162b2 XBB.1.5) specifically targeting the omicron-type SARS-CoV-2 XBB.1.5 subvariant. Therefore, COVID-19 vaccination (BNT162b2 XBB.1.5) tailored to this subvariant is recommended in all countries. However, similar to BNT162b2 vaccination, serious side effects such as shock, anaphylaxis, myocarditis, and pericarditis are associated with BNT162b2 XBB.1.5 vaccination [10].

Moreover, the occurrence of side effects after vaccination with mRNA-based COVID-19 vaccines is reported to be higher among Japanese individuals compared to Western populations [11]. Therefore, we conducted a retrospective observational clinical study to examine the levels of anti-omicron-type SARS-CoV-2 IgG and IgA in Japanese pregnant women who received the mRNA-based COVID-19 vaccine (BNT162b2 XBB.1.5), known for its efficacy against omicron-type SARS-CoV-2 variants. We compared the antibody titers of anti-SARS-CoV-2 spike glycoprotein IgG and IgA generated by vaccination in maternal serum, as well as the antibody levels in umbilical cord blood after vaccination with BNT162b2 XBB.1.5.

## 2. Materials and Methods

### 2.1. Sample Collection and Enzyme-Linked Immunosorbent Assay (ELISA)

This study included individuals who received the BNT162b2 XBB.1.5 vaccine (Pfizer-BioNTech), targeting the omicron-type SARS-CoV-2 XBB.1.5 subvariant, during pregnancy, specifically between 20 and 32 weeks’ gestation. Participants were recruited from a prospective study conducted across 28 academic medical centers in Japan, with their infants subsequently included in this follow-up study conducted from 20 September 2023 to 20 January 2024. Individuals who were infected before vaccination were excluded from this study.

Matched maternal and umbilical cord serum samples were collected at birth. For infants born to vaccinated mothers, capillary serum samples were obtained using a microneedle device at 2 months after birth, while samples for infants born to mothers who were vaccinated and those who had been infected with SARS-CoV-2 were collected at 6 months post-birth. All participants received two doses of the BNT162b2 XBB.1.5 vaccine administered 21 days apart.

Serum samples were collected before vaccine administration and then weekly for 6 weeks, starting from week 2 after the first dose. These samples were stored frozen until analysis. IgG levels were assessed using the Elecsys Anti-SARS-CoV-2 S serology assay and analyzed on the Cobas e801 analyzer, with a level of more than 0.8 U/mL considered positive (La Roche Ltd., Basel, Switzerland). IgA levels were measured using the EUROIMMUN AG Anti-SARS-CoV-2 S Kit, and an extinction ratio of samples over calibrator of more than 0.8 was considered positive (Methods in Supplementary Materials).

Human serum samples collected from maternal blood and umbilical cord blood were used for the detection of SARS-CoV-2 antibodies, specifically IgA and IgG, employing ELISA kits. For IgA analysis, the samples were initially diluted at a ratio of 1:25 in the appropriate sample dilution buffers. Subsequently, they were assessed using a semi-quantitative analysis ELISA kit following the manufacturer’s instructions (EUROIMMUN AG, Luebeck, Germany). In this assay, the microplate wells were coated with recombinant spike structural protein derived from the omicron-type SARS-CoV-2 XBB.1.5 subvariant. The results were interpreted by calculating a ratio of the extinction of samples over the extinction of the internal calibrator, with a ratio greater than 0.8 being considered positive. For IgG analysis, we used the Roche Elecsys Anti-SARS-CoV-2 S quantitative serology assay on the Roche Cobas e801 Analyzer according to the manufacturer’s instructions (La Roche Ltd., Basel, Switzerland). A result exceeding 0.8 units/mL, based on internal calibration curves, was considered positive (Methods in Supplementary Materials).

### 2.2. Clinical Research

We conducted a multi-center retrospective observational clinical study involving 148 participating pregnant women and 149 infants (including one set of twins), who underwent genomic medicine at genome medical facilities in Kyoto University Hospital, affiliated hospitals, and the National Hospital Organization Kyoto Medical Center in Japan. This study was reviewed and approved by the Central Ethics Review Board of the National Hospital Organization Headquarters in Japan (Approval code NHO R4-04) on 18 November 2020, and by the Kyoto University School of Medicine (Approval code M237) on 24 August 2022.

### 2.3. Informed Consent

All participants provided informed consent to participate in this study. Our clinical research adheres to the principles outlined in the Helsinki Statement.

### 2.4. Institutional Review Board Approval

Institutional Review Board Statement and Consent to Participate: The experiments involving the genome information of human pregnant women and fetuses obtained from genomic gene panels were conducted at Kyoto University, affiliated hospitals, and the National Hospital Organization Kyoto Medical Center in accordance with institutional guidelines.

This study was reviewed and approved by the Central Ethics Review Board of the National Hospital Organization Headquarters in Japan (Approval code NHO R4-04) on 18 November 2020, and by the Kyoto University School of Medicine (Approval code M237) on 24 August 2022.

### 2.5. Ethical Compliance with Participants

This manuscript contains personal and/or medical information about identifiable individuals, including a case report/case history. All authors have confirmed that this manuscript is sufficiently anonymized according to our anonymization policy, and consent was obtained from the participants. This study involves human participants and was approved by Ethics Committees and Institutional Boards. There is no involvement of animals in this study.

The authors underwent research ethics education through the Education for Research Ethics and Integrity (APRIN e-learning program, eAPRIN), with the following completion numbers for the authors: AP0000151756, AP0000151757, AP0000151769, and AP000351128. Consent to participate was required as this research was considered clinical research.

### 2.6. Statistical Analysis

All continuous data are presented as the mean with the standard error of the mean. Normality was assessed using the Shapiro–Wilk test. For comparing two groups, either the unpaired two-tailed *t*-test or the Mann–Whitney *U* test were used. Multiple comparisons were performed using either a one-way analysis of variance followed by a Tukey post hoc test or a Kruskal–Wallis analysis followed by a post hoc Steel–Dwass or Steel test. A *p*-value of less than 0.05 was considered statistically significant. All statistical analyses were conducted using JMP software (SAS Institute, Cary, NC, USA).

### 2.7. Data Availability

The data supporting the findings of this study are available from the corresponding author upon reasonable request.

Details of Materials and Methods are provided in Supplementary Materials, which are accessible online.

## 3. Results

**Figure 1:**
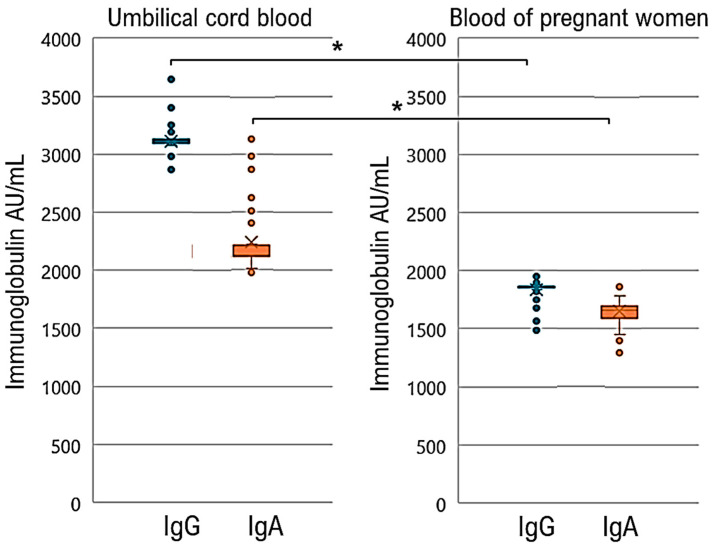
Comparison of the levels of anti-spike glycoprotein of SARS-CoV-2 immunoglobulin A (IgA) and immunoglobulin G (IgG) antibodies in maternal blood and umbilical cord blood during childbirth. The anti-SARS-CoV-2 IgA and IgG titers in umbilical cord blood were significantly higher than those in maternal blood. * *p* < 0.05.

Two doses of the omicron-type coronavirus vaccine (BNT162b2 XBB.1.5) administered to pregnant women may transfer antibodies to their babies. We measured the levels of neutralizing antibodies against SARS-CoV-2 in postnatal blood and cord blood obtained from the umbilical cords of 148 pregnant women who had received two doses of the vaccine. We observed that the level of neutralizing antibodies against SARS-CoV-2 in the umbilical cord blood (IgG Median 3115.8 U/mL, IgA Median 2234.5 U/mL) was 1.696 times and 1.353 times higher, respectively, than that in the mother’s blood (IgG Median 1837.3 AU/mL, IgA Median 1651.0 AU/mL) (Figure 1, Supplementary Table S1). Additionally, findings from a clinical study conducted in the United States supported these observations, showing that the level of neutralizing antibodies against SARS-CoV-2 in the umbilical cord blood (IgG Median 2170 AU/mL) was 1.069 times higher than that in the mother’s blood (IgG Median 2030 AU/mL) [12].

In a clinical study conducted by our facility in Japan, although the cohort was small, there is a possibility that, compared to the amount of neutralizing antibodies against SARS-CoV-2 in the cord blood of American pregnant women who received two doses of the BNT162b2 XBB.1.5 vaccine [12], the neutralizing antibodies produced in the bodies of Japanese pregnant women who received the same vaccine are more readily transferred to the umbilical cord blood. This finding suggests that anti-SARS-CoV-2 neutralizing antibodies produced in vaccinated pregnant women may be transferred to the fetus through the placenta.

**Figure 2:**
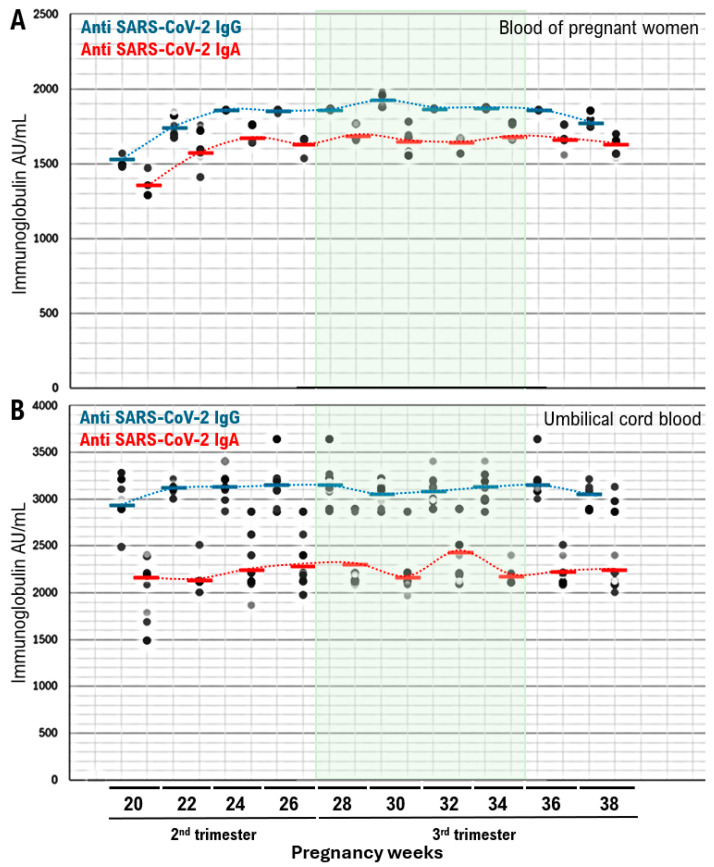
Anti-spike glycoprotein immunoglobulin A (IgA) and immunoglobulin G (IgG) titers in maternal blood and umbilical cord blood obtained from pregnant women vaccinated with the BNT162b2 XBB.1.5 vaccine, shown by gestational age at the time of vaccination. (**A**) Correlation between the time interval from the second vaccine dose and anti-SARS-CoV-2 IgA and IgG antibody levels in the blood of pregnant women. (**B**) Correlation between the time interval from the second vaccine dose and anti-SARS-CoV-2 IgA and IgG antibody levels in umbilical cord blood.

Additionally, we investigated the optimal timing during pregnancy for administering the second dose of the BNT162b2 XBB.1.5 vaccine to achieve the highest levels of neutralizing antibodies against SARS-CoV-2 transferable from the placenta. We found that the period of pregnancy with the highest levels of neutralizing antibodies (IgG Median 3320 AU/mL, IgA Median 2632.5 AU/mL) was between 28 and 34 weeks of gestation (Figure 2, Supplementary Table S1). Nonetheless, even in pregnant women who received the BNT162b2 XBB.1.5 vaccine during early pregnancy or close to childbirth, a relatively high level of neutralizing antibodies against SARS-CoV-2 were transferred to the fetus through the placenta.

Demographic and clinical characteristics are presented in Table 1. We defined severe neonatal morbidity (SNM) using an adaptation of the validated composite Neonatal Adverse Outcome Indicator, which includes 15 diagnoses and 7 procedures during the birth admission or within the first 28 days after birth. The risks of SNM, neonatal death, and Neonatal Intensive Care Unit admission were lower in infants of mothers vaccinated with BNT162b2 XBB.1.5 during pregnancy compared to those of unvaccinated mothers (Supplementary Table S2); notably, after inverse probability of treatment weighting, these significantly lower risks persisted. Systemic adverse effects following the first and second vaccine doses are indicated in Supplementary Table S3. Systemic side effects postvaccination are common symptoms observed in women of the same age who are not pregnant.

When a pregnant woman received the BNT162b2 XBB.1.5 vaccine during the third trimester of pregnancy, the titer of anti-SARS-CoV-2 antibodies in both maternal and umbilical cord blood at the time of delivery was high. Notably, there was no correlation between gestational age, neonatal weight, gender, or systemic adverse effects following BNT162b2 XBB.1.5 vaccination and the anti-SARS-CoV-2 antibody titers in maternal and umbilical cord blood at delivery (Supplementary Tables S2 and S3). Therefore, Japan’s Ministry of Health, Labor, and Welfare, in alignment with the U.S. National Institutes of Health, recommends BNT162b2 XBB.1.5 vaccination for pregnant women to prevent the severity of COVID-19 caused by SARS-CoV-2 infection in infants.

## 4. Discussion

A clinical study revealed the presence of neutralizing antibodies against the spike glycoprotein of SARS-CoV-2 in umbilical cord blood and breast milk from pregnant women vaccinated with the mRNA-based COVID-19 vaccine (BNT162b2) [13,14,15,16,17,18]. However, the spike glycoprotein of SARS-CoV-2 undergoes rapid mutations [19], leading to the emergence of numerous mutant viruses and their subvariants. Among these variants, the omicron-type SARS-CoV-2, which emerged in South Africa in November 2021, has demonstrated the ability to evade the immune response induced by vaccination with the mRNA-based COVID-19 vaccine (BNT162b2) [20,21]. Consequently, in Japan and other countries, vaccination with the BNT162b2 XBB.1.5 vaccine, tailored to the omicron SARS-CoV-2 spike glycoprotein, is recommended [22]. Aligning with these recommendations, we demonstrated that pregnant women vaccinated with BNT162b2 XBB.1.5 exhibit the transfer of neutralizing antibodies (IgG and IgA) against the spike glycoprotein of SARS-CoV-2 to both umbilical cord blood and breast milk [13,16,17,18].

A previous study reported that 96% of 27 pregnant women who received the COVID-19 vaccine tested positive for SARS-CoV-2 IgG antibodies at delivery [13]. Additionally, infants born to women vaccinated at least 3 weeks before delivery also tested positive for SARS-CoV-2 IgG antibodies [13]. This reinforces the positive correlation observed between anti-SARS-CoV-2 spike glycoprotein IgG levels in maternal blood post BNT162b2 XBB.1.5 vaccination and those in umbilical cord blood, consistent with previous findings. The efficacy of mRNA-based COVID-19 vaccination, such as BNT162b2, in reducing the severity of COVID-19 has been documented [19,20,21]. Consequently, research into vaccinating pregnant women, particularly those at high risk of severe COVID-19, is crucial for mitigating its impact on maternal health. The purpose of COVID-19 vaccination in pregnant women is to minimize disease severity in both the mother and fetus by ensuring adequate levels of anti-SARS-CoV-2 antibodies during pregnancy. Passive immunization through vaccination is pivotal in shielding the fetus from placental transmission of SARS-CoV-2 [23,24,25].

Our clinical study revealed that the level of neutralizing antibodies against SARS-CoV-2 in umbilical cord blood (3120 AU/mL) surpassed that in maternal blood (1860 AU/mL) by 1.68 times. Similarly, a study in the United States found that neutralizing antibody levels in umbilical cord blood (2170 AU/mL) were 1.05 times higher than those in maternal blood (2070 AU/mL) [10]. Additionally, a study in Israel assessed neutralizing antibodies against SARS-CoV-2 in pregnant women vaccinated during the second trimester and their newborns. The antibody titer in the newborn was 2.6 times higher than that in maternal blood [11], indicating that COVID-19 vaccination during the second trimester causes elevated antibody levels in mothers and newborns. These findings highlighted the importance of early vaccination for maternal and fetal health [13]. Differences in anti-SARS-CoV-2 spike glycoprotein neutralizing antibody titers between maternal and umbilical cord blood in clinical studies across different countries may be attributed to variations in the gestational weeks and ages of pregnant women at the time of COVID-19 vaccine administration. Furthermore, studies have indicated that if a vaccinated pregnant woman contracts SARS-CoV-2, the titer of anti-SARS-CoV-2 neutralizing antibodies in her blood may further increase [26].

In this clinical study, we assessed the levels of anti-spike glycoprotein antibodies against SARS-CoV-2, including IgG and IgA, in the blood samples of pregnant women and their umbilical cord blood. Administration of the BNT162b2 XBB.1.5 vaccine during the second half of pregnancy (28–34 gestational weeks) resulted in the highest levels of these antibodies in maternal blood. This immune response correlated with the transfer of neutralizing antibodies against SARS-CoV-2 to cord blood after vaccination. These findings support the recommendation for COVID-19 vaccination during the second trimester of pregnancy to ensure maternal and neonatal safety during the pandemic. However, our clinical trials included a limited number of pregnant participants. Therefore, conducting larger-scale clinical studies with a more extensive cohort of pregnant women is necessary to validate these findings.

## Figures and Tables

**Table 1:** Demographic and clinical characteristics of pregnant women vaccinated with the BNT162b2 XBB.1.5 mRNA COVID-19 vaccine.

Characteristics	Women (N = 148) ^a^
Maternal age, mean (SD), years	36.9 (4.9)
Body mass index, median (IQR) ^b^	26.5 (23.9–30.1)
Underlying systemic disease ^c^	29 (19.86)
Japanese	145 (99.31)
White people	1 (0.68)
Primipara	59 (40.41)
Multipara (2 births)	108 (73.97)
Grand multipara (5 births)	4 (2.74)
Systemic adverse effects after first vaccination ^d^	35 (23.97)
Systemic adverse effects after second vaccination ^d^	36 (24.32)
Gestational age at second vaccine dose, mean (5D), week	25.6 (3.5)
Systemic adverse effects ^d^	
After second vaccination	63 (43.15)
After first or second vaccination	68 (46.58)
Gestational age at birth, mean (SD), week	39.1 (1.4)
Duration from second vaccination to birth, mean (SD), week	14.4 (3.1)
SARS-CoV-2 lgG, IgA antibody level, median (range), AU/mL	
lntrapartum maternal IgG, IgA	IgG 3115.8 (2867–3403) IgA 2237.5 (1970–3128)
Umbilical cord blood IgG, IgA	IgG 1837.34 (1490–1954) IgA 1651.04 (1290–1780)
Newborn sex	
Male	77 (52.73)
Female	69 (47.27)
Newborn weight, mean (SD), g	3145.3 (412.6)

^a^ Data are presented as number (percentage) unless otherwise indicated. ^b^ Calculated as weight in kilograms divided by height in meters squared. ^c^ Hypertension (5 women), diabetes (6 women), asthma (4 women), thyroid disease (7 women), celiac (2 women), Crohn disease (1 woman), familial Mediterranean fever (1 woman), multiple sclerosis (2 women), and epidermolysis bullosa (1 woman). ^d^ General weakness, dizziness, fever, headache, general muscle aches, fatigue, and general rash.

## Data Availability

Data are available on various websites and have also been made publicly available (more information can be found in the first paragraph of the Results section). The transparency document associated with this article can be found in the online version at https://kyoto.hosp.go.jp/html/guide/medicalinfo/clinicalresearch/expand/gan.html (accessed on 18 May 2024).

## Supplementary Materials

The following supporting information can be downloaded at: https://www.mdpi.com/article/10.3390/idr16030036/s1, Table S1. Anti-Spike glycoprotein SARS-CoV-2 antibody (IgA, IgG) titer in blood and umbilical cord blood of pregnant women by week of pregnancy; Table S2. Association between COVID-19 vaccination BNT162b2 XBB.1.5 during pregnancy and neonatal and infant outcomes. Table S3. 28-day risk of adverse events following vaccination with COVID-19 vaccination: BNT162b2 XBB.1.5.

## Abbreviations

COVID-19: Coronavirus infectious disease, emerged in 2019; NIH: National Institution Health, IgG: immunoglobulin G, SARS-CoV-2: severe acute respiratory syndrome coronavirus-2.
