# Supplementary material for: High Transferability of Neutralizing Antibodies against SARS-CoV-2 to Umbilical Cord Blood in Pregnant Women Vaccinated with BNT162b2 XBB.1.5: A Retrospective Cohort Study
Source: Infect Dis Rep. 2024 May 27;16(3):481–90. doi: 10.3390/idr16030036 (PMC11203347; doi:10.3390/idr16030036)
Supplement: Supplementary file 1 [file idr-16-00036-s001.zip › eMethod with highlight ENG 05.22.2024.pdf]

Supplemental Online Content

High transferability of neutralizing antibodies against SARS-CoV-2 to umbilical cord blood in pregnant women with BNT162b2 XBB.1.5 vaccine

Hayashi T, Sano K, Konishi I. High transferability of neutralizing antibodies against SARS-CoV-2 to umbilical cord blood in pregnant women with BNT162b2 XBB.1.5 vaccine.

doi:

eMethods.

This supplemental material has been provided by the authors to give readers additional information about their work.

eMethods

Study design and participant selection

Pregnant individuals were eligible for inclusion in the prospective longitudinal pregnancy cohort (KMC IRB #2019-001108a) if they were 20 or older; received the available COVID-19 mRNA vaccine (BNT162b2 XBB.1.5) during pregnancy; were able to provide informed consent; and delivered at either National Hospital Organization Kyoto Medical Center and 28 affiliated medical facilities participating in clinical research.

Individuals with a history of SARS-CoV-2 infection prior to vaccination were excluded. Matched maternal and umbilical cord sera were collected during the delivery hospitalization. Liveborn infants of enrolled pregnant participants were enrolled after birth (KMC IRB #2019-001108b).

In a cross-sectional study design, we sought to assess infant titers at 2 months or 6 months of life, as these are clinically relevant time points when pediatric vaccines may be given, and there may be theoretical concern about persistence of infant titer resulting in antibody interference with vaccine response. Infants born to pregnant individuals testing positive for SARS-CoV-2 between 20 and 32 weeks' gestation were enrolled as a comparator group for antibody transfer. Due to the timing of the ninth wave of the COVID-19 pandemic in Japan (late spring to early winter 2023) relative to the period of sample collection (early winter and early spring 2024), we could only capture the 4-month timepoint in infants born to mothers received the BNT162b2 XBB.1.5 vaccine (Pfizer-BioNTech).

In Japan, the Ministry of Health, Labor and Welfare reports that people who are pregnant, breastfeeding, or planning to become pregnant can also receive the COVID-19 vaccines. In Japan, there are no reports that the COVID-19 vaccines approved in Japan have any adverse effects on pregnancy, fetuses, breast milk, or reproductive organs. Japan's Ministry of Health, Labor and Welfare recommends that pregnant women receive the COVID-19 vaccine based on the Immunization Law.

In Japan, as it becomes difficult to consider the difference between the development of congenital abnormalities in babies unrelated to COVID19 vaccination and the development of congenital abnormalities in babies due to COVID19 vaccination, it is recommended that pregnant women avoid vaccination up to 12 weeks of pregnancy.

In our clinical study, pregnant women who were infected with SARS-CoV-2 (positive SARS-CoV-2 test) and had symptoms of COVID19 before vaccination with the BNT162b2 XBB.1.5 vaccine were excluded from the cohort.

Sample* collection *and processing

Maternal and umbilical cord sera were collected via venipuncture (National Hospital Organization Kyoto Medical Center, Kyoto, MA) during delivery hospitalization. Serum separator tubes were centrifuged at 1000 g for 10 minutes at room temperature. Serum and plasma were aliquoted into cryogenic vials and stored at -80°C.

Enzyme-linked immunosorbent assay (ELISA)

Antibodies against the SARS-CoV-2 Spike glycoprotein were quantified using an ELISA. ELISA plates were coated with 500 ng/mL of SARS-CoV-2 XBB.1.5 (Omicron) Spike protein S1+S2 trimer Protein (His) expressed in HEK293 mammalian cells with His tag (the predicted molecular weight is 136.27 kDa, TargetMol Chemicals Inc. Boston, MA, US) and incubated for 30 minutes at room temperature. Plates were washed with washing buffer (0.05% Tween-20, 400mM NaCl, 50mM Tris, pH 8.0) and blocked with a 0.1% Bovine Serum Albumin (BSA)

solution for 30 minutes at room temperature. Plates were washed, and sample was added at a dilution of 1:100. Plates were incubated with sample at 37°C for 30 minutes. Plates were washed, and a horseradish peroxidase (HRP)-conjugated goat anti-human IgG antibody or HRP conjugated goat anti-human IgA antibody (Bethyl Laboratories, Montgomery, Texas, USA) was added for detection of Spike-specific IgG and Spike-specific IgA. Plates were incubated with secondary antibody for 30 minutes at room temperature and then washed. 3,3',5,5'-tetramethylbenzidine (TMB) was used to develop the ELISA () and sulfuric acid was used to stop the ELISA. Signal was read at 450 nm and Phosphate Buffered Saline (PBS)-background corrected from a reference wavelength of 570 nm. Detectable anti-Sike glycoprotein IgG or anti-Sike glycoprotein IgA was defined as any value greater than the sum of the mean value of assay negative controls (known SARS-CoV-2 negative, unvaccinated individuals) and 3 x the standard deviation of those samples. The ELISA kit used in this experiment is shown below. IgG levels were detected by the Elecsys Anti–SARS-CoV-2 S serology assay and read on the Cobas e801 analyzer with a level of more than 0.8 U/mL considered positive (La Roche Ltd) and IgA with the EUROIMMUN AG Anti-SARS-CoV-2 S Kit with an extinction ratio of samples over calibrator of more than 0.8 considered positive.

Statistical Analyses

Differences between vaccinated and naturally-infected groups depicted in Table 1 were assessed using Mann Whitney U test for continuous variables, or Fisher's exact test for categorical variables. P<0.05 was considered significant. Statistical analyses were performed using R, version 4.0.2, and Graphpad Prism v.9.0.

eTable S2. Association Between COVID-19 Vaccination: BNT162b2 XBB.1.5 During Pregnancy and Neonatal and Infant Outcomes

	No./total No. (%)
Outcome	Vaccine exposed
Severe neonatal morbidity	1/149* (0.67)
Neonatal death	0/149 (0.00)
Neonatal intensive care unit (NICU) admission	2/149 (1.34)
Neonatal readmission	2/149 (1.34)
Discharged within 7 day after birth	2/149 (1.34)
Hospital admission up to 6 month of age	0/149 (0.00)
Discharged within 7 day after birth	0/149 (0.00)

*A pregnant woman who received the COVID-19 vaccine gave birth to twins.

eTable S3. 28-Day Risk of Adverse Events Following Vaccination With COVID-19 Vaccination: BNT162b2 XBB.1.5

	BNT162b2 XBB.1.5 vaccine (risk period)
Outcome	No. of events/person-years
Ischemic cardiac event	1/148 (0.67%)
Any cerebrovascular event	0/148 (0.00%)
Cerebral infarction only (including TIA)	0/148 (0.00%)
Arterial thromboembolism	0/148 (0.00%)
Deep vein thrombosis	0/148 (0.00%)
Pulmonary embolism	0/148 (0.00%)
Myocarditis	0/148 (0.00%)
Pericarditis	0/148 (0.00%)
Cerebral venous thrombosis	0/148 (0.00%)
Thrombocytopenia and coagulative disorders	1/148 (0.67%)
Guillain−Barre syndrome	0/148 (0.00%)
Bell palsy	0/148 (0.00%)
Transverse myelitis	0/148 (0.00%)
Encephalomyelitis or encephalitis	0/148 (0.00%)
Narcolepsy	2/148 (1.35%)
Appendicitis	0/148 (0.00%)
Aseptic arthritis	1/148 (0.67%)
Type 1 diabetes	0/148 (0.00%)
Subacute thyroiditis	1/148 (0.67%)
Heart failure	0/148 (0.00%)
Arrhythmia	3/148 (2.03%)
Acute liver failure	0/148 (0.00%)
Acute kidney failure	0/148 (0.00%)
Acute pancreatitis	0/148 (0.00%)
Erythema multiforme	1/148 (0.67%)
Seizure	2/148 (1.35%)
Arterial aneurysm	0/148 (0.00%)
Uveitis	2/148 (0.67%)
Muscle pain	38/148 (25.68%)
Arthro joint pain	34/148 (22.97%)
Fever	22/148 (14.86%)
Fatigue	93/148 (62.84%)
Headache	81/148 (54.73%)
Chills	47/148 (31.76%)
Diarrhea	3/148 (2,20%)
